# A rare case of giant hepatic angiomyolipoma with subcapsular rupture

**DOI:** 10.3389/fsurg.2023.1164613

**Published:** 2023-07-10

**Authors:** Cuiting Wu, Yang Yang, Feng Tian, Yuan Xu, Qiang Qu

**Affiliations:** ^1^ Department of General Surgery, Peking Union Medical College Hospital, Chinese Academy of Medical Science and Peking Union Medical College, Beijing, China; ^2^ Department of Orthopedic, Peking Union Medical College Hospital, Chinese Academy of Medical Science and Peking Union Medical College, Beijing, China; ^3^ Department of Thoracic Surgery, Peking Union Medical College Hospital, Chinese Academy of Medical Science and Peking Union Medical College, Beijing, China

**Keywords:** hepatic angiomyolipoma (HAML), subcapsular rupture, laparoscopic—laparotomy, left liver, case report

## Abstract

Hepatic angiomyolipoma (HAML) is a rare mesenchymal neoplasm that predominantly affects middle-aged women. In this study, we present a case of a 49-year-old woman with a giant HAML accompanied by spontaneous subcapsular rupture. The patient initially experienced nausea and abdominal distention, followed by an enlargement of the upper abdominal circumference. Laboratory examination revealed decreased serum hemoglobin, while tumor biomarkers were within normal ranges. Imaging studies, such as abdominal ultrasound and contrast-enhanced computed tomography (CT), demonstrated a large upper abdominal mass with heterogeneous density and hypervascularity. The tumor appeared to have invaded the left liver, raising concerns about possible malignancy. Subsequent positron-emission tomography/CT confirmed increased fluorodeoxyglucose uptake in the mass. Laparoscopic exploration revealed a protruding, well-encapsulated tumor from the left liver, exhibiting subcapsular hemorrhage. Surgical resection of the tumor and the left liver was performed, leading to a successful outcome.

## Introduction

Hepatic angiomyolipoma (HAML) is a rare hepatic mesenchymal neoplasm first reported in 1976 (1). It is generally composed of blood vessels and smooth muscle cells, and adipose tissue, with great variety in proportions (2). A review published in 2016 identified 292 patients with one or more HAML, and most of them are middle-aged women (1). Calame et al. (3) identified 10 HAML patients who had a spontaneous rupture in 2021. Here, we present a rare case of a patient who had a giant HAML with spontaneous subcapsular rupture.

## Case report

**Figure 1:**
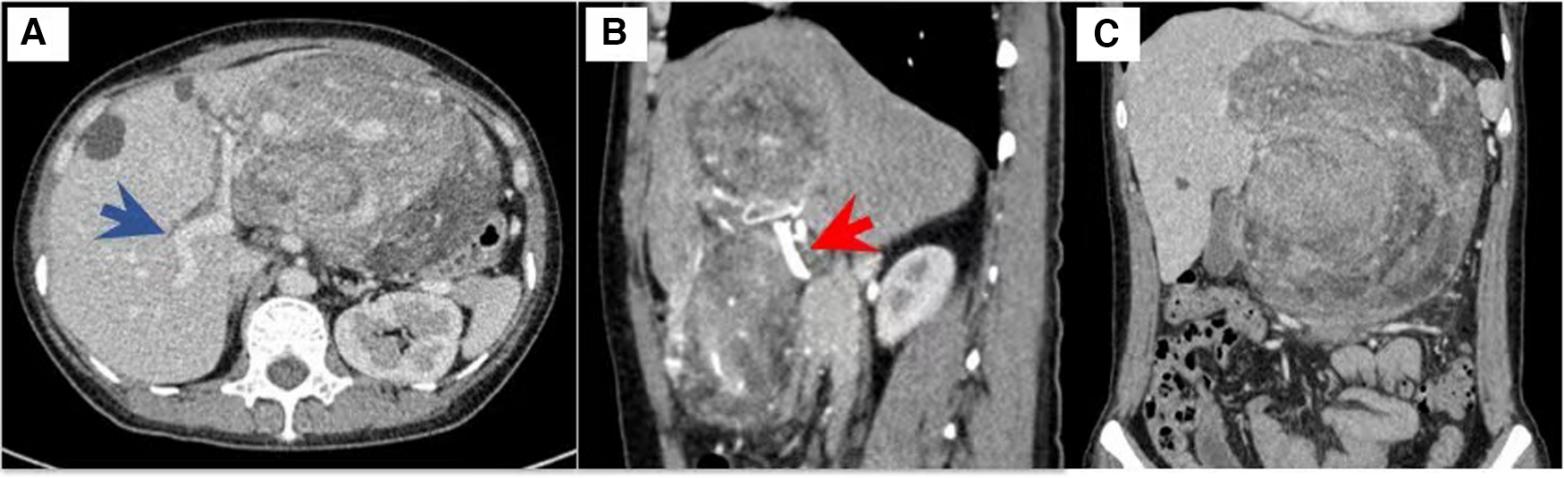
Contrast-enhanced computed tomography of the mass. Blue arrow, portal vein. Red arrow, hepatic artery.

A 49-year-old woman presented with a 6-month history of nausea and abdominal distention after meals, followed by an enlargement of the upper abdominal circumference. She had no symptoms of jaundice, abdominal pain, or constipation. Her laboratory examination showed decreased serum hemoglobin (78 g/L) and normal white blood cell counts. Tumor biomarkers, such as alpha-fetoprotein (AFP), carcinoembryonic antigen (CEA), carbohydrate antigen 19-9 (CA19-9), and carbohydrate antigen 125 (CA125), were all negative. Abdominal ultrasound showed a slightly hyperechoic mass in the upper abdominal region, exhibiting echo heterogenicity, without obvious blood flow signal. Contrast-enhanced computed tomography (CT) revealed a 15 cm × 10 cm upper abdominal mass with heterogeneous density (Figure 1, dot lines: maximal size of the mass, 18.4 cm × 16.2 cm). The risk of malignancy is indicated by the mass potential invasion into the left liver. Blood supply was shown to come from the celiac trunk. The middle hepatic vein was compressed, and the left hepatic vein was not revealed. Positron-emission tomography (PET)/CT revealed a slightly increased ^18^F-fluorodeoxyglucose (FDG) intake, with a standardized uptake value (SUV) max of 2.4.

Retroperitoneal tumor, gastrointestinal stromal tumor, and left hepatic tumor were suspected before surgery. Hepatic hemangioma is the most common benign hepatic tumor, characterized by a hypervascular appearance on imaging studies such as contrast-enhanced CT or magnetic resonance imaging (MRI). However, in contrast to HAML, hemangiomas typically demonstrate a homogeneous enhancement pattern during the arterial phase, followed by a progressive centripetal filling and delayed washout during the venous phase. Furthermore, the absence of a central scar and the presence of fat components in HAML can aid in differentiating it from hepatic hemangioma.

**Figure 2:**
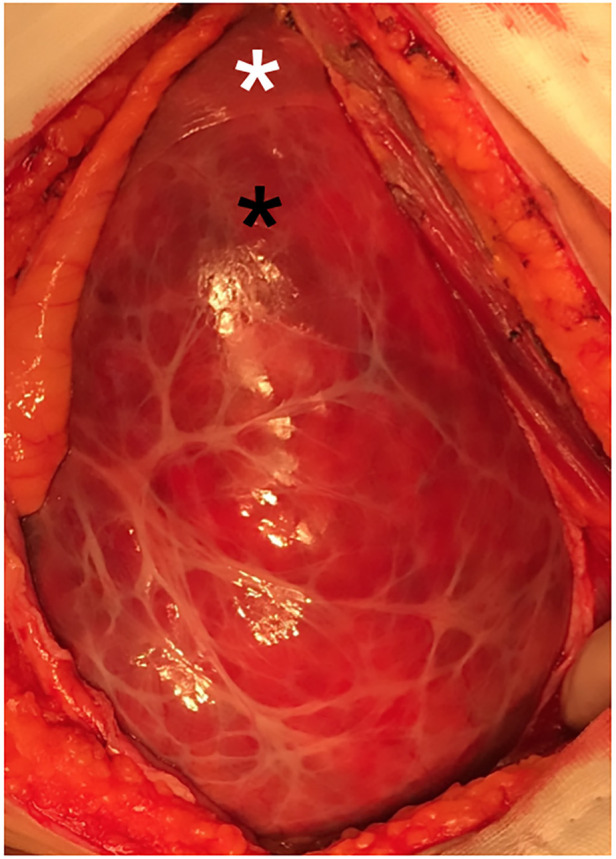
Intraoperative image of the mass. white asterisk: left liver; black asterisk: the tumor with subcapsular hemorrhage.

As the preoperative diagnosis was uncertain, there was a risk associated with puncture due to potential hemorrhage. Therefore, we decided to proceed directly with surgical intervention. During laparoscopic exploration, a protruding, well-encapsulated tumor from the left liver was identified. A laparoscopic approach was employed for the resection of the tumor and left liver. Subcapsular hemorrhage indicated a spontaneous rupture (Figure 2, white asterisk, the left liver; black asterisk, the tumor with subcapsular hemorrhage). Thus, the entire tumor and left liver were resected. This patient recovered well and was discharged on postoperative day 6.

**Figure 3:**
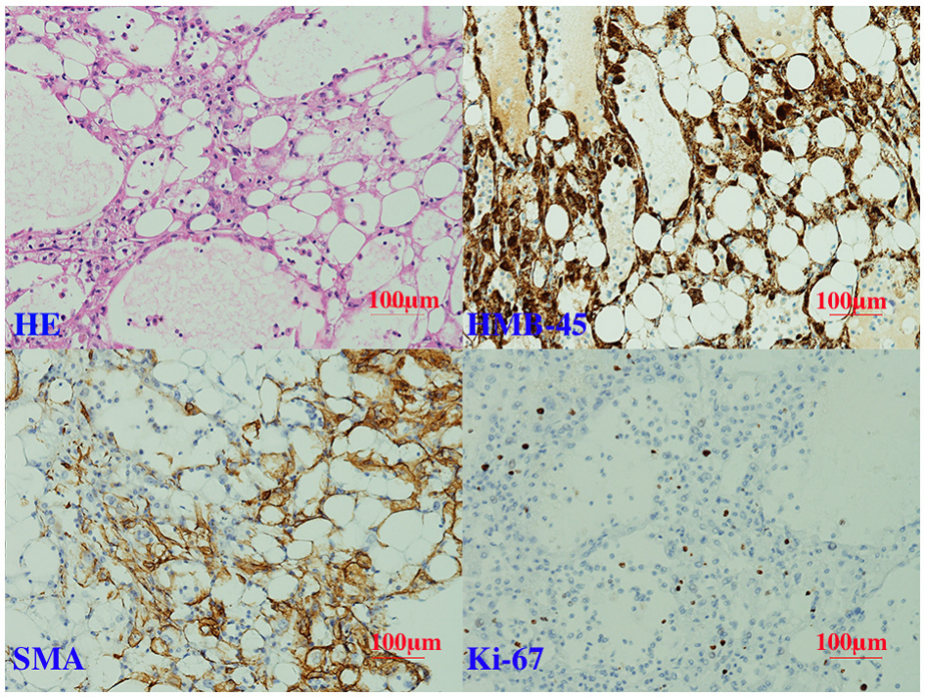
Histological examination of the mass.

Her histological examination (Figure 3) showed that the tumor was composed of mixed proportions of blood vessels, smooth muscle cells (SMCs), and adipose tissue. Immunohistochemistry staining indicated that the tumor had positive homatropine methylbromide-45 (HMB-45), smooth muscle actin (SMA), and Melan-A (not shown) but was negative for S100. The Ki-67 index was 5%.

## Discussion

HAML typically occurs in a non-cirrhotic liver. Generally, patients with HAML have no obvious symptoms and find the tumor occasionally during medical examination (42%–72%) (4). In renal AML, tumor size (≥4 cm) and pregnancy are known risk factors for rupture (5). However, the risk factors for the rupture of HAML remain unclear, given the paucity of available data. In previous studies, the size of ruptured HAML was often reported as small (with a median of 4 cm) (3). Only one case of HAML with a size greater than 10 cm (12 cm) had been reported (6). In this report, the size of HAML is 18 cm. Despite the subcapsular rupture, the capsular is still intact. Current evidence suggests that tumor size may not be a risk factor for rupture. However, more evidence is needed to draw a convincing conclusion.

HAML is typically difficult to diagnose before biopsy due to its significantly varying composition. It was summarized that only 28.2% of patients had the correct diagnosis before resection (1). In this case, there was an increase in the degree of malignancy suspicion due to the adjacent tissues being heavily compressed, which strongly resembled an “invasion” as seen on CT.

Although HAML is typically regarded as benign, its aggressive potential is drawing more and more attention as evidence increases. Thus, radical resection should be considered for suspected patients, especially taking into consideration that the tumor has a risk of rupture. Some risk factors, e.g., tumor size greater than 5 cm, infiltrative growth pattern, high nuclear grade, necrosis, and mitotic activity >1/50 high power field, were proposed (3). This patient needs further surveillance for a better prognosis. The limitation of this case report is that this is a single-patient design and further studies with larger patient cohorts are needed to validate our observations.

## Data Availability

The original contributions presented in the study are included in the article, further inquiries can be directed to the corresponding author.
